# Distribution and prevalence of malaria parasites among long-tailed macaques (*Macaca fascicularis*) in regional populations across Southeast Asia

**DOI:** 10.1186/s12936-016-1494-0

**Published:** 2016-09-02

**Authors:** Xinjun Zhang, Khamisah Abdul Kadir, Leslie Fabiola Quintanilla-Zariñan, Jason Villano, Paul Houghton, Hongli Du, Balbir Singh, David Glenn Smith

**Affiliations:** 1 Department of Anthropology, University of California Davis, One Shields Avenue, Davis, CA 95616 USA; 2 Malaria Research Centre, Universiti Malaysia Sarawak, 94300 Kota Samarahan, Sarawak Malaysia; 3 Unit for Laboratory Animal Medicine, University of Michigan Medical School, 2800 Plymouth Rd., Ann Arbor, MI 48109 USA; 4 Primate Products, Inc., PO Box 1588, Immokalee, FL 34143 USA; 5 School of Bioscience and Bioengineering, South China University of Technology, Guangzhou, 510006 China

**Keywords:** *Plasmodium knowlesi*, *Plasmodium cynomolgi*, *Macaca fascicularis*, Geographic distribution, Biased infection rate

## Abstract

**Background:**

*Plasmodium knowlesi* and *Plasmodium cynomolgi* are two malaria parasites naturally transmissible between humans and wild macaque through mosquito vectors, while *Plasmodium inui* can be experimentally transmitted from macaques to humans. One of their major natural hosts, the long-tailed macaque (*Macaca fascicularis*), is host to two other species of *Plasmodium* (*Plasmodium fieldi* and *Plasmodium coatneyi*) and is widely distributed in Southeast Asia. This study aims to determine the distribution of wild macaques infected with malarial parasites by examining samples derived from seven populations in five countries across Southeast Asia.

**Methods:**

*Plasmodium knowlesi, P. cynomolgi, P. coatneyi, P. inui* and *P. fieldi*, were detected using nested PCR assays in DNA samples from 276 wild-caught long-tailed macaques. These samples had been derived from macaques captured at seven locations, two each in the Philippines (n = 68) and Indonesia (n = 70), and one each in Cambodia (n = 54), Singapore (n = 40) and Laos (n = 44). The results were compared with previous studies of malaria parasites in long-tailed macaques from other locations in Southeast Asia. Fisher exact test and Chi square test were used to examine the geographic bias of the distribution of *Plasmodium* species in the macaque populations.

**Results:**

Out of 276 samples tested, 177 were *Plasmodium*-positive, with *P. cynomolgi* being the most common and widely distributed among all long-tailed macaque populations (53.3 %) and occurring in all populations examined, followed by *P. coatneyi* (20.4 %)*, P. inui* (12.3 %)*, P. fieldi* (3.4 %) and *P. knowlesi* (0.4 %). One *P. knowlesi* infection was detected in a macaque from Laos, representing the first documented case of *P. knowlesi* in wildlife in Laos. Chi square test showed three of the five parasites (*P. knowlesi*, *P. coatneyi*, *P. cynomolgi)* with significant bias in prevalence towards macaques from Malaysian Borneo, Cambodia, and Southern Sumatra, respectively.

**Conclusions:**

The prevalence of malaria parasites, including those that are transmissible to humans, varied among all sampled regional populations of long-tailed macaques in Southeast Asia. The new discovery of *P. knowlesi* infection in Laos, and the high prevalence of *P. cynomolgi* infections in wild macaques in general, indicate the strong need of public advocacy in related countries.

## Background

Most malaria parasites are considered to be host-specific, in that most of them infect only one host species, though a single host can be infected by multiple *Plasmodium* species [1]. Humans are the natural hosts for four *Plasmodium* species: *Plasmodium falciparum, Plasmodium malariae, Plasmodium vivax*, and *Plasmodium ovale* [2]. Since 1930, three other species of *Plasmodium* that typically infect wild macaques in Southeast Asia had been known to be transmissible to humans. This was first demonstrated by passage with blood infected with *Plasmodium knowlesi* [3], and later with *Plasmodium cynomolgi* [4] and *Plasmodium inui* [5]. Furthermore, accidental infections of humans in research laboratories with *P. cynomolgi* through mosquito bites were reported in 1960 [4, 6] and 1980 [7], and also human infections through mosquito-transmission experiments in the 1960s with *P. cynomolgi* [4, 6, 8] and *P. inui* [9].

In 1965, the first case of a human naturally infected with knowlesi malaria was reported [10] and human infections in nature were considered extremely rare until in 2004, when 120 of 208 malaria patients at Kapit Hospital in Sarawak, Malaysian Borneo were found to be infected with *P. knowlesi* using nested PCR assays [11]. Additional human knowlesi malaria cases were subsequently reported throughout Southeast Asian countries, including Thailand, Singapore, Peninsular Malaysia, Cambodia, Vietnam, Indonesia and the Philippines [12], and in the Nicabar and Andaman Islands of India [13]. In 2014, another malaria parasite of long-tailed macaques, *P. cynomolgi*, was reported to have naturally infected a woman in Peninsular Malaysia [14]. These recent discoveries of humans infected with what were previously thought to be non-human malarias implied the potential larger distribution of human cases that may have not yet been confirmed and highlighted the potentially high risks of more zoonotic malaria infections.

The natural hosts of *P. knowlesi* and *P. cynomolgi* are long-tailed macaques (*Macaca fascicularis*) and pig-tailed macaques (*Macaca nemestrina*) [2]. Long-tailed macaques are widely distributed in Southeast Asia and exhibit the third most widespread geographical distribution among all primates after humans and rhesus macaques (*Macaca mulatta*) [15]. Their natural range extends southward and eastward from Southeastern Bangladesh and Burma, through the Southern part of the Indochinese Peninsula (Thailand, Cambodia, Laos, and Vietnam), and into the Malaysian Peninsula including Singapore, and the islands of Sumatra, Borneo, Java, and the Philippines [16].

Other than *P. knowlesi* and *P. cynomolgi*, long-tailed macaques are also natural hosts of three other *Plasmodium* species, namely *P. coatneyi, P. fieldi* and *P. inui* [11, 17]. The prevalence of these three parasites and their distribution in regional long-tailed macaque populations, however, had only been characterized for macaques in Malaysia and Singapore [18–20]. Other studies have examined blood samples from macaques by either molecular detection assays solely for *P. knowlesi* in Thailand, Peninsular Malaysia and Indonesia [21–25], or by sequencing of the *Plasmodium* mitochondrial genome in Malaysian Borneo [26]. A thorough investigation of *Plasmodium* infections in long-tailed macaques is necessary to determine their potential as a public health threat. The present study utilizes sensitive molecular detection methods, and characterizes the prevalence of five *Plasmodium* species in seven long-tailed macaque populations across the geographic range of these non-human primates in Southeast Asia.

## Methods

### DNA samples

**Fig. 1:**
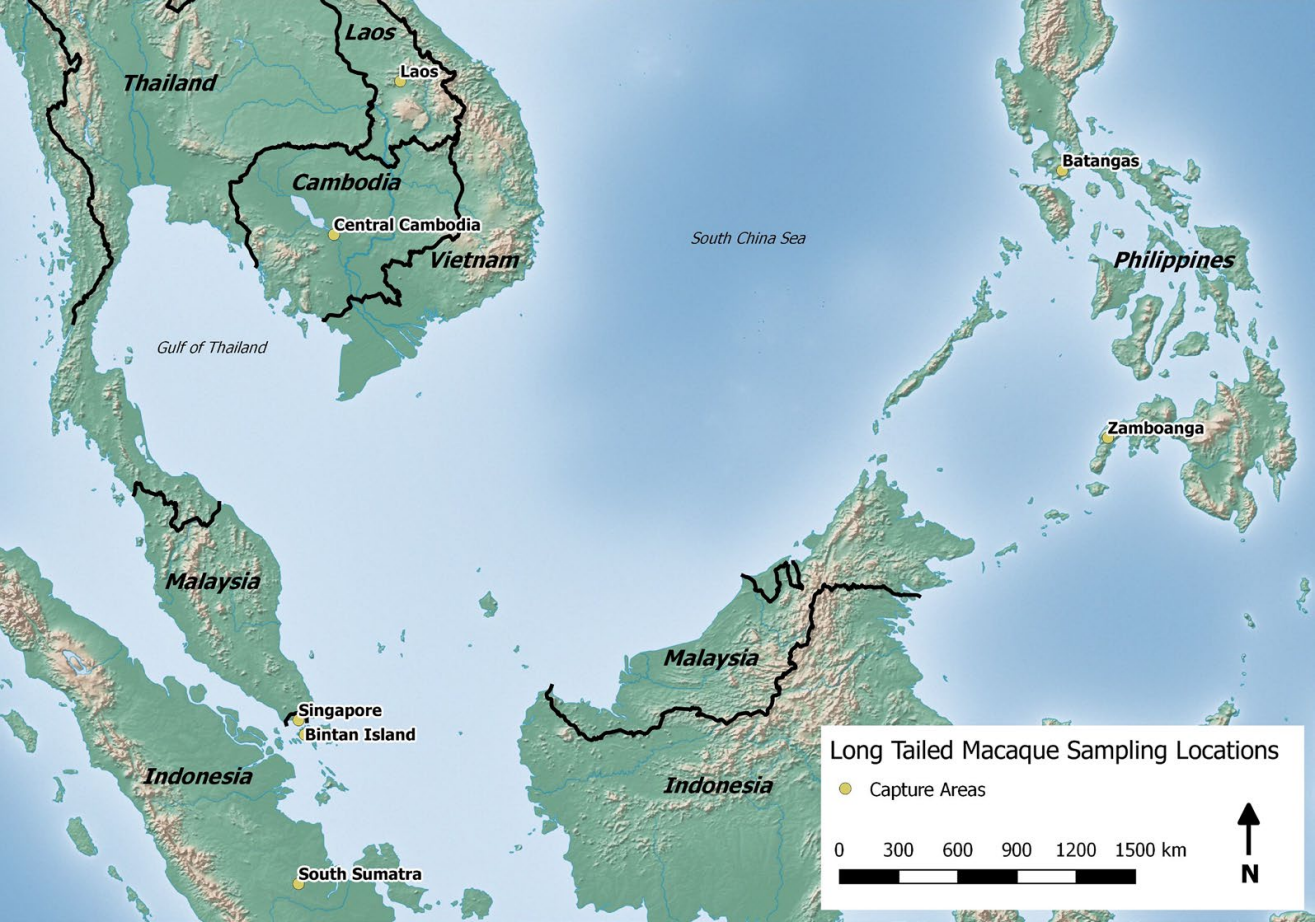
Locations of origin of capture of long-tailed macaques

DNA samples of 276 wild-caught long-tailed macaques from seven populations in Southeast Asian countries were employed for this study. These samples had been collected for genetic studies that were reported elsewhere [27–29]. These included 50 samples from Southern Sumatra, 54 samples from Cambodia, 40 samples from Zamboanga in Southern Philippines, 28 samples from Batangas in Northern Philippines, 40 samples from Singapore, 20 samples from Bintan island in Indonesia (near Singapore), and 44 samples from Laos. These DNA samples were obtained from single time point blood collection and were stored at −20 °C in the Molecular Anthropology Laboratory at the University of California, Davis. DNA samples were obtained from long-tailed macaques that were all originally wild-caught. A map illustrating the geographic distribution and the origins of these samples is shown as Fig. 1.

### Nested PCR assays

Malaria parasites have distinct small subunit ribosomal RNA (SSU rRNA) genes that are developmentally regulated [30]. Nested PCR assays targeting the distinct SSU rRNA genes of *Plasmodium* species were used to amplify DNA from macaque blood samples [31]. Samples were initially examined using *Plasmodium*-specific primers and those that were positive were subsequently examined with species-specific primers. The PCR parameters and primers used to detect *Plasmodium*, *P. knowlesi, P. cynomolgi, P. inui, P. fieldi* and *P. coatneyi* have been described previously [21, 32]. Plasmids containing the SSU rRNA genes of *P. knowlesi, P. cynomolgi, P. inui, P. fieldi* and *P. coatneyi* were used as positive controls and were provided by the Malaria Research Centre at Universiti Malaysia Sarawak [19]. The amplification products were analyzed by gel electrophoresis and data of *Plasmodium* infections of the samples were compared with published data in *Plasmodium* infections from wild long-tailed macaques in Sarawak, Malaysian Borneo, and multiple sites from Peninsular Malaysia, Thailand, Singapore and Indonesia [19–26].

### Sequencing of PCR amplified fragment and sequence analysis

One of the PCR amplified fragments, generated from a macaque from Laos with *P. knowlesi*-specific primers, was sequenced using the Sanger dideoxy sequencing method [33] and analyzed on the ABI 3730 Capillary Electrophoresis Genetic Analyzer. The DNA sequences were aligned by Clustal W and analysed using DNASTAR software (Lasergene, USA) and DnaSP version 4.10.8. The phylogenetic analysis was inferred by the Neighbor-joining method [34] with Kimura 2-parameter, including transitions and transversions, using MEGA version 3.1. The reliability of the internal nodes of the tree was assessed by the bootstrap method after 1000 replicates.

### Analysis of prevalence of malaria parasites

To determine whether the prevalence of each *Plasmodium* species is geographically biased, Fisher exact test was performed on software R (version 3.2.4) on the distribution table. The null hypothesis under the Fisher exact test is, “prevalence and region are independent, i.e., prevalence of malaria parasite does not change as geographic location changes”. For the test, 10^7^ random tables with the same column and row totals as the observed data, assuming row and column independence, were generated. The *P* value cited is the sum of the probabilities of all generated tables that have individual probabilities smaller than those in the observed table. To compare each observed value with its expected value under null hypothesis, Chi square tests were performed in R on the observed table to generate each expected value under the null hypothesis.

## Results

Out of 276 long-tailed macaques screened for *Plasmodium* DNA using nested PCR assays, 177 were found to be positive (Table 1). Multiple infections with different species were observed in each of 32 samples. Among the positive samples, the frequency of single, double and triple infections was 81.9, 17.0, and 1.1 %, respectively. *Plasmodium cynomolgi* was detected in at least one macaque from all seven sampled locations, showing the highest prevalence (53.3 %) and most widespread distribution among macaques across Southeast Asia, followed by *P. coatneyi* (20.4 %), *P. inui* (12.3 %), *P. fieldi* (3.4 %), and *P. knowlesi* (0.4 %). The prevalence of each parasite species among the macaque populations varied by location (Table 2; Fig. 1), and these were compared with previous published studies conducted in Malaysian Borneo [21, 28], Thailand [24, 25], Peninsular Malaysia [20, 27], Singapore [22], and Indonesia [26].

**Table 1:** Malaria parasite infections in long-tailed macaques by location

Infection	*Plasmodium* species	Number of long-tailed macaques infected							
		Laos	Singapore	Vanny, Cambodia	Batangas, Philippines	Zamboanga, Philippines	Southern Sumatra	Bintan Island	Total
Single	Pk	1							1
	Pcy	28	23	24	2	1	39	9	126
	Pct			7		1		1	9
	Pin		2	1		2	1	2	8
	Pfi		1						1
Double	Pcy, Pin		1	2			9	2	15
	Pin, Pfi		1						1
	Pct, Pfi	1	1						2
	Pcy, Pfi			1				1	2
	Pct, Pcy		1		1				1
	Pin, Pct			9					9
Triple	Pcy, Pin, Pfi		1					1	2
Total *Plasmodium*-positive		30	31	44	3	4	49	16	177 (64.1 %)
Total *Plasmodium*-negative		14	9	10	25	36	1	4	99 (35.9 %)
Total number of macaques		44	40	54	28	40	50	20	276 (100 %)

*Pk P. knowlesi, Pcy P. cynomolgi, Pin P. inui*, *Pct P. coatneyi, Pfi P. fieldi*

**Table 2:** Comparison of prevalence of species of *Plasmodium* in long-tailed macaques

Sample origin	Total number	No. Pk (χ^2^ Exp.)	% Pk	No. Pcy (χ^2^ Exp.)	% Pcy	No. Pin (χ^2^ Exp.)	% Pin	No. Pct (χ^2^ Exp.)	% Pct	No. Pfi (χ^2^ Exp.)	% Pfi	Year
Batangas, Philippines	28	0 (1)	0	3 (2)	10.7	0 (1)	0	1 (1)	3.6	0 (0)	0	2012
Zamboanga, Philippines	40	0 (1)	0	1 (2)	2.5	2 (1)	5	1 (1)	2.5	0 (0)	0	2012
Vanny, Cambodia	54	0 (12)	0	27 (22)	50	12 (12)	22.2	16 (8)	29.6	1 (2)	1.9	2011
Southern Sumatra	50	0 (13)	0	48 (23)	96	10 (12)	20	0 (8)	0	0 (2)	0	2010
Guidong/Laos	44	1 (7)	2.3	29 (12)	65.9	0 (6)	0	0 (4)	0	1 (1)	2.3	2013
Bintan Island, Indonesia	20	0 (5)	0	13 (8)	65	5 (4)	25	1 (3)	5	2 (1)	10	2007
Singapore	40	0 (8)	0	26 (15)	65	5 (8)	12.5	2 (5)	5	4 (1)	10	2007
Total	276	1	0.4	147	53.3	34	12.3		20.4	12	3.4	
Sarawak, Malaysian Borneo [21]	82	71 (54)	86.6	52 (99)	63.4	69 (51)	84.1	52 (35)	63.4	4 (9)	4.9	2011
Sabah, Malaysian Borneo [26]	26	4 (4)	15.4	3 (7)	11.5	8 (4)	30.8	1 (2)	3.8	1 (1)	3.8	2014
Singapore [22]	157	45 (21)	28.7	40 (39)	25.5	1 (20)	0.6	2 (14)	1.3	11 (4)	7	2011
Hulu Selangor, Malaysia [20]	70	21 (17)	30.0	18 (31)	25.7	23 (16)	32.9	16 (11)	22.9	1 (3)	1.4	2015
Kuala Lumpur and Selangor, Malaysia [27]	41	0	0	−	−	−	−	−	−	−	−	2008
Kuala Lipis, Pahang, Malaysia [27]	75	10	13.3	−	−	−	−	−	−	−	−	2008
Thailand [25]	195	1	0.5	−	−	7	3.6	3	1.5 %	0	0	2010
Thailand [24]	105	0	0	−	−	−	−	−	−	−	−	2008
Indonesia [26]	N/A	N/A	3.2 %	−	−	−	−	−	−	−	−	2007

The figures in brackets are expected number of infections estimated by Chi square test under the assumption of “*Plasmodium* parasite prevalence independent from the geographic location”

*Pk P. knowlesi, Pcy P. cynomolgi*, *Pin P. inui, Pct P. coatneyi, Pfi P. fieldi, No* number, *χ* ^*2*^ *Exp*. Chi square test expected values, *−* not done, *N/A* not available

The *P* value for the Fisher exact test is 10^−7^, indicating that the observed geographical distribution of *Plasmodium* infection is extremely unlikely under the null hypothesis; i.e., the prevalence of each species of *Plasmodium* exhibits bias among different geographic locations.

According to the comparison between observed number of infected macaques with what is expected by the Chi square test under the null hypothesis (Table 2), *P. inui* and *P. fieldi* both exhibited relatively uniform distributions over geographical locations. The prevalence of *P. knowlesi* and *P. cynomolgi* in macaques from Sarawak is higher and lower, respectively than all other sites. The prevalence of *P. knowlesi* in macaques from all other locations is lower than expected. A much higher prevalence of *P. cynomolgi* in the macaque samples from Southern Sumatra and Singapore was also observed, along with relatively higher prevalence of *P. coatneyi* in macaques from Sarawak and Cambodia, and lower prevalence of *P. coatneyi* in macaques from Sumatra and Laos. Macaques from Bintan Island and Singapore, two locations in close geographical proximity, showed close similarities in the infection profiles. In addition, the prevalence for all *Plasmodium* species were unusually low in the macaques from the Philippines compared to those from other geographic regions.

**Fig. 2:**
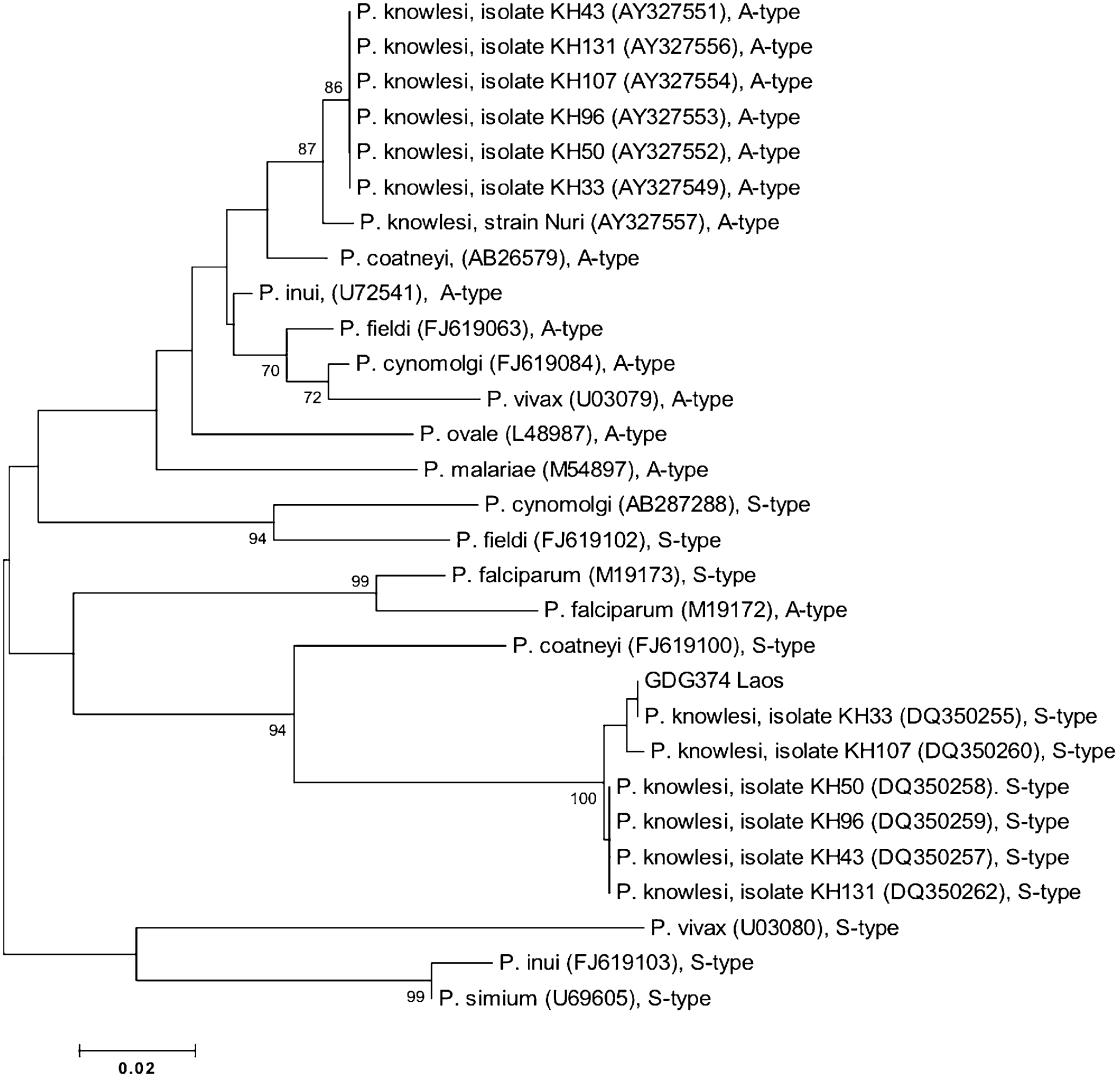
Phylogenetic tree based on the SSU rRNA genes of Plasmodium species produced by the Neighbor-joining method. Accession numbers for each species of Plasmodium are stated within brackets; Only bootstrap values above 70 % are shown; ‘S-type’ = sexually transcribed form and ‘A-type’ = asexually transcribed form

Only one sample from Laos was positive for *P. knowlesi*. The nested PCR assay with *P. knowlesi*-specific primers gave a similar result for this sample when the assay was repeated, using new nest 1 and nest 2 PCR amplifications. The sequence of the SSU rRNA gene fragment amplified in the nested PCR assay was aligned and compared with *Plasmodium* SSU rRNA sequences in GenBank [21]. It was identical with the sequence of the S-type SSU rRNA gene of *P. knowlesi* isolate KH33, and 99.6 % similar with sequences from *P. knowlesi* isolates KH50, KH96, KH107, KH43 and KH 131. Phylogenetic analysis confirmed that the macaque from Laos was infected with *P. knowlesi* (Fig. 2).

## Discussion

Long-tailed macaques are widely distributed in both suburban areas and dense forests in Southeast Asia [15]. Historically and culturally, long-tailed macaques particularly have an extensive interface with humans, especially locals who share spatial overlap with them and also tourists who come in contact with them. The close contact and geographic range overlap between humans and long-tailed macaques introduce to both species a situation of mutual disease transmission. Humans in Southeast Asian countries who are naturally exposed to long-tailed macaques are at potential risks of being infected with a number of simian pathogens, including simian T cell lymphotropic viruses (STLV), simian retrovirus (SRV), *Macacine herpesvirus 1* (B virus), and the zoonotic malarias [35–38]. Understanding the distribution patterns of macaques that carry malaria parasites especially the ones transmissible to humans facilitates an effective assessment of this human health threat.

The present study demonstrates that two macaque malaria parasites naturally transmissible to humans, *P. cynomolgi*, and *P. inui*, exist among all the 7 sampled regional populations of wild long-tailed macaques. Specifically, *P. cynomolgi* showed distinctively and uniformly high prevalence in the macaques, except for the two populations from the Philippines. The low prevalence of *Plasmodium* in macaques from Zamboanga in the southern Philippines and Batangas in the northern Philippines does not suggest that host genetic differences are responsible, because long-tailed macaques in the latter geographic region are assigned to a uniquely different subspecies (*M. f. philippensis*) than those (*M. f. fascicularis*) inhabiting all other regions studied [29]. It is only recently confirmed that *P. cynomolgi* can also be transmitted to humans naturally and thus represents a potential new zoonotic malaria [14]. The observation of the high prevalence of *P. cynomolgi* in macaques from this study indicates the strong need of public advocacy in the affected areas.

A complex nature of *Plasmodium* infections in natural hosts was noted in this study. Among the 177 malaria-positive macaques, 18.1 % were each found to have multiple (double and triple) *Plasmodium* species infections. More multiple infections, including co-infections with four or five species of *Plasmodium* in each long-tailed macaque were reported in other studies [19, 20]. This complex nature of infections represents challenges for accurate identification by microscopy, as well as a potential underestimation of prevalence and distribution of *Plasmodium* parasites in macaques and other primates.

In previous studies, it was shown that in peri-domestic and urban areas of Singapore, Peninsular Malaysia, and Thailand, the parasitaemia and the presence of *Plasmodium* infections in free-ranging macaques were low or zero, hypothetically due to the absence or limited presence of known competent vectors of malaria parasites [17, 25, 39]. A comparison with multiple studies using molecular methods to detect *Plasmodium* infections in macaques from other locations indicates that the prevalence of malaria and of the different species of *Plasmodium* vary according to the sampling site, even on a small island like Singapore [18–20, 22–26]. Comparing the Singaporean samples screened from this study, which were collected from 2007, with the Singaporean samples reported from 2011 [20], there was a decrease in the prevalence of *P. knowlesi* infection. The exact location from where the macaques were trapped in Singapore is not known for the current study and the differences in the prevalence of *P. knowlesi* between the two studies could be due to different sampling locations in Singapore. In addition, in a study conducted in Sabah, Malaysian Borneo, Fornace et al. [40] have shown spatial heterogeneity in *P. knowlesi* incidence depending on the land type. They found that the incidence of *P. knowlesi* varied between village, forests, and historical forest loss areas, which further indicates that environmental changes influence the transmission of *P. knowlesi*. More recently, Moyes and co-workers used a newly developed incidence–environment assessment model to investigate the distributions of reservoir hosts and Anopheline vectors of *P. knowlesi* in Southeast Asia and assessed their relationships with the environment [41]. Their analyses showed that the predicted distribution of both the long-tailed macaques and vectors of knowlesi malaria encompassed a wide range of habitats, most significantly in the northern part of their study area which included Myanmar, Thailand, Laos, Cambodia and Vietnam. These recent studies highlight that environmental variables possibly are the key driving force in determining the distribution of *Plasmodium* parasites.

It has been noted that the parasitaemia of malaria parasites in a human or non-human primate host could change on a daily basis [2]. Because the DNA samples used in this study were from single time points, further studies are warranted to investigate and follow up the change of infections over time in macaques.

The detection of *P. knowlesi* infection from a long-tailed macaque from Laos is the first report of *P. knowlesi* infection in either humans or macaques in that country. This finding should encourage further investigations of *P. knowlesi* using molecular detection methods among macaques and humans in Laos.

## Conclusions

The prevalence of malaria parasites that naturally infect long-tailed macaques, including those that are transmissible to humans, varies among regional populations of wild long-tailed macaques in Southeast Asia. *Plasmodium cynomolgi*, which can be naturally acquired by humans, exists in all wild long-tailed macaque populations studied with varying prevalence. This is the first report of a natural infection of *P. knowlesi* in a macaque from Laos. The presence of malaria parasites underscores the risk of potential zoonotic infections in the local human populations.

## Abbreviations

PCR: Polymerase chain reaction
Nest1: Nested PCR amplification round 1
Nest2: Nested PCR amplification round 2
SSU rRNA: Small subunit ribosomal RNA
STLV: Simian T cell lymphotropic viruses
SRV: Simian retrovirus

## References

[1] Garnham PCC (1966). Malaria parasites and other haemosporidia.

[2] Coatney GR, Collins WE, Warren M, Contacos PG. The primate malarias. U.S. Government Printing Office. 1971.

[3] Knowles R, Gupta B (1932). A study of monkey-malaria, and its experimental transmission to man. Indian Med Gaz.

[4] Eyles DE, Coatney GR, Getz ME (1960). Vivax-type parasite of macaques transmissible to man. Science.

[5] Das Gupta BM (1938). Transmission of *P. inui* to man. Proc Natl Inst Sci India.

[6] Schmidt LH, Greenland R, Genther CS (1960). The transmission of *Plasmodium cynomolgi* to man. Am J Trop Med Hyg.

[7] Druilhe P, Trape JF, Leroy JP, Godard C, Gentilini M (1980). Deux cas d’infection humaine accidentelle par *Plasmodium cynomolgi bastianellii*. Etude clinique sérologique. Ann Soc Belge Med Trop.

[8] Coatney GR, Elder HA, Contacos PG, Getz ME, Greenland R, Rossan RN (1961). Transmission of the M strain of *Plasmodium cynomolgi* to man. Am J Trop Med Hyg.

[9] Coatney GR, Chin W, Contacos PG, King HK (1966). *Plasmodium inui*, a quartan-type malaria parasite of Old World monkeys transmissible to man. J Parasitol.

[10] Chin W, Contacos PG, Coatney GR, Kimball HR (1965). A naturally acquired quotidian-type malaria in man transferable to monkeys. Science.

[11] Singh B, Sung LK, Matusop A, Radhakrishnan A, Shamsul SSG, Cox-Singh J (2004). A large focus of naturally acquired *Plasmodium knowlesi* infections in human beings. Lancet.

[12] Singh B, Daneshvar C (2013). Human infections and detection of *Plasmodium knowlesi*. Clin Microbiol Rev.

[13] Tyagi RK, Das MK, Singh SS, Sharma YD (2013). Discordance in drug resistance-associated mutation patterns in marker genes of *Plasmodium falciparum* and *Plasmodium knowlesi* during coinfections. J Antimicrob Chemother.

[14] Ta TH, Hisam S, Lanza M, Jiram AI, Ismail N, Rubio JM (2014). First case of a naturally acquired human infection with *Plasmodium cynomolgi*. Malar J.

[15] Fooden J (1995). Systematic review of Southeast Asian longtail macaques, *Macaca fascicularis* (Raffles, 1821). Fieldiana Zool.

[16] Eudey AA (2008). The crab-eating macaque (*Macaca fascicularis*): widespread and rapidly declining. Primate Conserv.

[17] Jeslyn WPS, Huat TC, Vernon L, Irene LMZ, Sung LK, Jarrod LP (2011). Molecular epidemiological investigation of *Plasmodium knowlesi* in humans and macaques in Singapore. Vector Borne Zoonotic Dis.

[18] Akter R, Vythilingam I, Khaw LT, Qvist R, Lim YAL, Sitam FT (2015). Simian malaria in wild macaques: first report from Hulu Selangor district, Selangor, Malaysia. Malar J.

[19] Lee KS, Divis PC, Zakaria SK, Matusop A, Julin RA, Conway DJ (2011). *Plasmodium knowlesi*: reservoir hosts and tracking the emergence in humans and macaques. PLoS Pathog.

[20] Li Meizhi I. Identification and molecular characterisation of simian malaria parasites in wild monkeys of Singapore. National University of Singapore, MSc thesis. 2011:184.

[21] Jongwutiwes S, Buppan P, Kosuvin R, Seethamchai S, Pattanawong U, Sirichaisinthop J (2011). *Plasmodium knowlesi* malaria in humans and macaques, Thailand. Emerg Infect Dis.

[22] Seethamchai S, Putaporntip C, Malaivijitnond S, Cui L, Jongwutiwes S (2008). Malaria and *Hepatocystis* species in wild macaques, southern Thailand. Am J Trop Med Hyg.

[23] Putaporntip C, Jongwutiwes S, Thongaree S, Seethamchai S, Grynberg P, Hughes AL (2010). Ecology of malaria parasites infecting Southeast Asian macaques: evidence from cytochrome b sequences. Mol Ecol.

[24] Jones-Engel L, Engel G, Schillaci M, Pacheco M, Escalante A (2007). Malarial monkeys: reservoir for zoonotic infection?. Am J Primatol.

[25] Vythilingam I, Noorazian YM, Huat TC, Jiram AI, Yusri YM, Azahari AH (2008). *Plasmodium knowlesi* in humans, macaques and mosquitoes in peninsular Malaysia. Parasit Vectors.

[26] Muehlenbein MP, Pacheco MA, Taylor JE, Prall SP, Ambu L, Nathan S (2015). Accelerated diversification of nonhuman primate malarias in Southeast Asia: adaptive radiation or geographic speciation?. Mol Biol Evol.

[27] Ng J, Trask JS, Houghton P, Smith DG, Kanthaswamy S (2015). Use of genome-wide heterospecific single-nucleotide polymorphisms to estimate linkage disequilibrium in Rhesus and *Cynomolgus macaques*. Comp Med.

[28] Kanthaswamy S, Ng J, Ross CT, Trask JS, Smith DG, Buffalo VS (2013). Identifying human-rhesus macaque gene orthologs using heterospecific SNP probes. Genomics.

[29] Smith DG, Ng J, George D, Trask JS, Houghton P, Singh B (2014). A genetic comparison of two alleged subspecies of Philippine cynomolgus macaques. Am J Phys Anthropol.

[30] Rogers MJ, Li J, McCutchan F, Sherman IW (1998). The Plasmodium rRNA genes: developmental regulation and drug target. Malaria: parasite biology, pathogenesis and protection.

[31] Singh B, Bobogare A, Cox-Singh J, Snounou G, Abdullah MS, Rahman HA (1999). A genus- and species-specific nested polymerase chain reaction malaria detection assay for epidemiologic studies. Am J Trop Med Hyg.

[32] Daneshvar C, Davis TME, Cox-Singh J, Rafa’ee MZ, Zakaria SK, Divis PCS (2009). Clinical and laboratory features of human *Plasmodium knowlesi* infection. Clin Infect Dis.

[33] Sanger F, Nicklen S, Coulson AR (1977). DNA sequencing with chain-terminating inhibitors. Proc Natl Acad Sci USA.

[34] Saitou NM (1987). The neighbor-joining method: a new method for reconstructing phylogenetic trees. Mol Biol Evol.

[35] Engel GA, Jones-engel L, Schillaci MA, Suaryana KG, Putra A, Fuentes A (2002). Human exposure to Herpesvirus B-seropositive macaques, Bali, Indonesia. Emerg Infect Dis.

[36] Fuentes A (2006). Human culture and monkey behavior: assessing the contexts of potential pathogen transmission between macaques and humans. Am J Primatol.

[37] Jones-Engel L, Engel GA, Schillaci MA, Rompis A, Putra A, Suaryana KG (2005). Primate-to-human retroviral transmission in Asia. Emerg Infect Dis.

[38] Wallis J, Lee DR (1999). Prevention of disease transmission in primate conservation. Int J Primatol.

[39] Putaporntip C, Hongsrimuang T, Seethamchai S, Kobasa T, Limkittikul K, Cui L (2009). Differential prevalence of *Plasmodium* infections and cryptic *Plasmodium knowlesi* malaria in humans in Thailand. J Infect Dis.

[40] Fornace KM, Abidin TR, Alexander N, Brock P, Grigg MJ, Murphy A (2016). Association between landscape factors and spatial patterns of *Plasmodium knowlesi* infections in Sabah, Malaysia. Emerg Infect Dis.

[41] Moyes CL, Shearer FM, Huang Z, Wiebe A, Gibson HS, Nijman V (2016). Predicting the geographical distributions of the macaque hosts and mosquito vectors of *Plasmodium knowlesi* malaria in forested and non-forested areas. Parasites Vectors.

